# Real‐world management of maple syrup urine disease (MSUD) metabolic decompensations with branched chain amino acid‐free formulas in France and Germany: A retrospective observational study

**DOI:** 10.1002/jmd2.12207

**Published:** 2021-03-06

**Authors:** Pascale de Lonlay, Roland Posset, Ulrike Mütze, Karine Mention, Delphine Lamireau, Manuel Schiff, Aude Servais, Jean Baptiste Arnoux, Anaïs Brassier, Myriam Dao, Claire Douillard, Chris Ottolenghi, Clément Pontoizeau, Federica Miotto, Jeannie Le Mouhaër

**Affiliations:** ^1^ Service et Centre de Référence des maladies métaboliques Hôpital Necker – Enfants Malades, APHP, Université de Paris, Filière G2M France; ^2^ Center for Pediatric and Adolescent Medicine, Division of Pediatric Neurology and Metabolic Medicine University Hospital Heidelberg Heidelberg Germany; ^3^ Unité Métabolisme et Centre de Référence Pôle Enfant, CHRU de Lille – Hôpital Jeanne de Flandre, Filière G2M Lille France; ^4^ Centre de compétence des maladies métaboliques CHU de Bordeaux‐GH Pellegrin, Filière G2M Bordeaux France; ^5^ Service d'endocrinologie‐diabétologie‐métabolisme‐nutrition Hôpital Huriez, CHRU Lille France; ^6^ Recordati Milan Italy; ^7^ Recordati Rare Diseases Puteaux France

**Keywords:** BCAA‐free formula, maple syrup urine disease, metabolic decompensations, observational study

## Abstract

Maple syrup urine disease (MSUD) is a rare inborn metabolic disorder, managed with a strict protein‐restricted diet. At any time or age patients may still experience metabolic decompensations, requiring administration of branched chain amino acid (BCAA)‐free formula to reduce leucine levels. This retrospective observational study of 126 decompensation episodes from 54 MSUD patients treated at five centers in France and Germany from 2010 to 2016, describes episodes and outcomes for patients stratified into groups who received enteral/oral or intravenous (IV) BCAA‐free formula, and by pediatric or adult age categories. IV administration of BCAA‐free formula was required in cases of gastric intolerance (33%), refusal to undergo nasogastric tubing (31%), “emergency” (14%) or coma patients (8%), and as prophylaxis before surgery (6%). Overall, mean duration of hospitalization was 6.6 days with oral/enteral BCAA‐free formula and 5.4 days with IV formula. Leucine levels at discharge decreased by a mean of 548.5 μmol/L (69.3%) in the oral/enteral group and 657.2 μmol/L (71.3%) in the IV group. In the pediatric subgroup, there were no marked differences between administration groups on any outcome. In the adult subgroup, mean time to episode resolution was 15.8 days in the oral/enteral group and 7.7 days in the IV group (*P* = .008); mean duration of hospitalization was 6 days in the oral/enteral group and 4.6 days in the IV group (*P* = NS). Overall, seven serious adverse events in two patients were reported, of which only nausea and vomiting were treatment related.

## 1 INTRODUCTION

Maple syrup urine disease (MSUD) is an autosomal recessive metabolic disease caused by a dysfunction in the branched‐chain α‐ketoacid dehydrogenase enzyme complex. The normal breakdown of branched‐chain amino acids (BCAA)—valine, isoleucine, and leucine—is absent or impeded.^1^ MSUD is rare, with an estimated incidence of 1 in 185 000‐491 000 births.^2^, ^3^ Although the clinical phenotype can vary in severity, generally MSUD is characterized by feeding difficulties soon after birth, which, if untreated, result in seizures, coma, development delays, and irreversible neurologic damage or death.^4^, ^5^, ^6^ The neurotoxicity is caused by accumulation of leucine and its metabolite, α‐ketoisocaproic acid,^7^, ^8^ which causes profound metabolic alterations and impairs energy homeostasis.^9^, ^10^, ^11^

If diagnosed and treated early, prognosis is generally good.^12^, ^13^ MSUD is managed by a protein‐restricted diet with BCAA‐free formula supplementation.^2^, ^4^ However, even patients who strictly adhere to dietary restrictions still experience metabolic decompensations.^14^ As decompensations can be triggered by physiological or even psychological stress, they can occur at any age.^12^ Metabolic decompensations require urgent therapy to normalize leucine levels to avoid permanent neurological damage or death.^11^, ^12^ Metabolic decompensations in MSUD are treated by providing a BCAA‐free formula to reduce plasma BCAA levels within reference ranges and to ensure that the leucine‐isoleucine‐valine balance is maintained.^15^, ^16^, ^17^ BCAA‐free formulas given during metabolic decompensations are provided at higher concentration than standard BCAA‐free formulas due to a further reduction of intact protein intake during metabolic decompensations. There is currently no specific drug for the treatment of decompensation episodes. The general treatment approach is to assess the severity of the episode based on both plasma leucine levels and clinical symptoms, which commonly include decreased appetite, nausea and vomiting, irritability and altered consciousness, and so on. BCAA‐free formula is available as a food supplement, administered orally or enterally. When oral or enteral administration is difficult or impossible (ie, in cases of vomiting, coma or seizures), or in case of refusal of nasogastric tube feeding, an intravenous (IV) formula may be necessary.^15^, ^16^, ^17^ Although not widely available in Europe, IV BCAA‐free formula has been used in France since 2010 for the treatment of metabolic decompensation episodes in emergency settings or in combination with or as an alternative to invasive extracorporeal procedures, such as hemodialysis and/or hemofiltration (HD/HF).^12^, ^13^, ^14^

Although some management recommendations are available,^15^ there are currently no European guidelines or approved medicinal products specifically for the treatment of MSUD decompensation episodes. The evidence regarding the real‐world management of MSUD decompensation is also limited, which has resulted in the emergence of heterogeneous standards of care across Europe. The aims of this study were: (a) to describe the real‐world management of metabolic decompensation episodes in a large cohort of MSUD patients hospitalized in centers in France and Germany and (b) to explore the management of such episodes with oral/enteral or IV BCAA‐free formulas in terms of laboratory and clinical outcomes.

## 2 METHODS

### 2.1 Study design

This is an observational, noninterventional retrospective study to provide insight into the clinical management of decompensation episodes. Data extrapolated from medical records of MSUD patients hospitalized for decompensation episodes between January 1, 2010 and December 31, 2016 were collected at four rare‐disease reference centers in France and one in Germany. The study was conducted according to the *Helsinki Declaration*, *Guidelines for Good Pharmacoepidemiology Practices*, and in accordance with French and German privacy laws. The study was approved by the University of Heidelberg Ethics Committee application no. S‐664/2018 and by the *CNIL* in France. Informed consent or assent was required from all patients and/or their legal guardians.

### 2.2 Data collection

Relevant clinical data relating to both MSUD and decompensation episodes were collected for each patient for up to three consecutive episodes in France or up to 10 consecutive episodes in Germany. More episodes were permitted at the German center to balance the number of episodes between France and Germany. The maximum limit was applied to include as many episodes as possible but without over‐representing patients with frequent episodes. Data confidentiality was ensured by pseudonymized patient records and only the investigational team on site were able to link the data to the corresponding patient.

### 2.3 Inclusion criteria

Patients were included with a biochemically confirmed diagnosis of MSUD and were under surveillance as per local practice at their reference center. Patients were required to have a fully documented medical history including: demographics, MSUD characteristics, clinical outcomes, and management details of each decompensation episode. Patients had to have experienced at least one decompensation episode requiring >24 hours of hospitalization with a plasma leucine >381 μmol/L at admission.

### 2.4 Definitions

“Decompensation episodes” were defined by plasma leucine levels above 381 μmol/L (5.0 mg/dL) at hospital admission. “Plasma leucine levels” were classified per established clinical protocol as: “normal”: <381 μmol/L; mild: [381‐762] μmol/L; “moderate”: [762‐1144] μmol/L; “severe”: [1144‐1525] μmol/L; or “critical”: ≥1525 μmol/L. “Baseline leucine” was the first reported leucine level ≥381 μmol/L at the time of admission. “Episode resolution” was defined as the patient achieving normalization of leucine levels (<381 μmol/L), together with resolution of symptoms. “Oral/enteral BCAA‐free formulas” were commercially available products and the names were not recorded, thus composition may have varied. “IV BCAA‐free solution” was supplied as a hospital preparation by the French General Agency of Equipment and Health Products (*AGEPS*) and administered at a standard dose of 1‐2 g/kg of body weight per day.^16^ Its composition is reported in Table S1. The IV BCAA‐free solution was not available at the German center.

Episodes were retrospectively stratified to two administration route groups: an “oral/enteral” group (n = 69, 54.8%), where episodes were initially treated with BCAA‐free formula either orally, enterally (via nasogastric tubing) or via gastrostomy, irrespective of a subsequent switch from oral to enteral or vice versa during the same episode, but excluding episodes (n = 5, 4.0%) for patients who switched to IV administration; and an “IV” group (n = 36, 28.6%), which included patients treated with IV BCAA‐free formula upon admission, irrespective of whether they subsequently received oral or enteral formula during the same episode. Sixteen episodes (n = 16, 12.7%) were treated with HD/HF. These episodes were included in the overall analysis but excluded from the exploratory one. One hundred and five episodes were re‐stratified at the time of episode onset, to a “pediatric” group (<18 years, n = 79) or an “adult” group (≥18 years, n = 26).

### 2.5 Statistical methods

All available relevant data were included in the analysis and no imputation was applied. Median survival times and the corresponding 95% confidence intervals (CI) were estimated by the Kaplan‐Meier method using a two‐sided log‐rank test. *P*‐values were determined by Wilcoxon rank‐sum test for continuous variables. A *P*‐value of <.05 was considered significant. As this was a descriptive study with heterogeneous study groups receiving varied treatments, statistical comparison testing was not considered clinically relevant and was only performed on the Kaplan‐Meier analyses of “time to episode resolution” for the pediatric and adult subgroups. The analyses were performed at episode level by sites and globally using SAS software version 9.4 (SAS Institute Inc., Cary, NC).

## 3 RESULTS

At study completion, data were available for 55 patients (27 female/28 male) and 129 episodes (Table 1).

**TABLE 1:** Patient and episodes characteristics by site

	Site 1 (n = 16)	Site 2 (n = 72)	Site 3 (n = 13)	Site 4 (n = 12)	Site 5 (n = 13)	Total episodes (n = 126)
Sex						
Male	10 (62.5%)	29 (40.3%)	7 (53.8%)	3 (25%)	11 (84.6%)	60 (47.6%)
Female	6 (37.5%)	43 (59.7%)	6 (46.1%)	9 (75.0%)	2 (15.4%)	66 (52.4%)
Mean (SD) age at episode onset (years)	7.7 (6.1)	11.4 (10)	16.3 (5.3)	4.2 (2.6)	6.0 (5.9)	10.2 (8.9)
Age at episode onset by age groups						
<1 year	2 (12.5%)	14 (19.4%)	0	1 (8.3%)	3 (23.1%)	20 (15.9%)
1–6 years	7 (43.8%)	12 (16.7%)	0	8 (66.7%)	5 (38.5%)	32 (25.4%)
6‐12 years	1 (6.3%)	15 (20.8%)	4 (30.8%)	3 (25.0%)	2 (15.4%)	25 (19.8%)
12‐18 years	5 (31.3%)	9 (12.5%)	4 (30.8%)	0	3 (23.1%)	21 (16.7%)
≥18 years	1 (6.3%)	22 (30.6%)	5 (38.5%)	0	0	28 (22.2%)
Precipitating conditions of acute decompensation episodes						
Febrile illness/Infection^a^	7 (43.8%)	27 (37.5%)	5 (38.5%)	8 (66.7%)	4 (30.8%)	51 (40.5%)
Hormonal changes/Menstruations	0	1 (1.4%)	0	0	0	1 (0.8%)
Surgery	1 (6.3%)	5 (6.9%)	0	0	2 (15.4%)	8 (6.3%)
Stress	1 (6.3%)	0	0	0	0	1 (0.8%)
Vaccination	1 (6.3%)	1 (1.4%)	0	0	0	2 (1.6%)
Diet issues^a^	3 (18.8%)	17 (23.6%)	6 (46.2%)	0	2 (15.4%)	28 (22.2%)
No obvious apparent cause	4 (25.0%)	22 (30.6%)	2 (15.4%)	3 (25%)	2 (15.4%)	33 (26.2%)
Other^b^	1 (6.3%)	6 (8.3%)	2 (15.4%)	1 (8.3%)	3 (23.1%)	13 (10.3%)
Presence of clinical manifestations	10 (62.5%)	49 (68.1%)	4 (30.8%)	12 (100%)	10 (76.9%)	85 (67.5%)
Emergency treatments						
BCAA‐free formula	16 (100%)	72 (100%)	13 (100%)	11 (91.7%)	13 (100%)	125 (99.2%)
Glucose/Dextrose	15 (93.8%)	62 (86.1%)	8 (61.5%)	3 (25%)	13 (100%)	101 (80.2%)
High caloric	16 (100%)	46 (63.9%)	6 (46.2%)	3 (25%)	13 (100%)	84 (66.7%)
Lipids	1 (6.3%)	61 (84.7%)	5 (38.5%)	2 (16.7%)	13 (100%)	82 (65.1%)
Administration of BCAA‐free formula during episode						
Enteral (tube)—Total	5 (31.3%)	41 (56.9%)	2 (15.4%)	9 (81.8%)	12 (92.3%)	69 (55.2%)
IV—Total	0	44 (61.1%)	4 (30.8%)	2 (18.2%)	1 (7.7%)	51 (40.8%)
Oral—Total	12 (75%)	38 (52.8%)	8 (61.5%)	2 (18.2%)	0 (0.00%)	60 (48.0%)
Gastrostomy—Total	0	2 (2.8%)	0	0	0	2 (1.6%)
Duration of BCAA‐free formula (hours)						
N	16 (100%)	32 (44.4%)	9 (69.2%)	10 (90.9%)	13 (100%)	80 (64%)
Mean (SD)	121.5 (66.8)	112.6 (107.2)	109 (68.6)	99.5 (46.8)	160.6 (194.6)	120.1 (110.4)
Median (range)	108 (48, 279)	81.0 (7, 600)	96 (24, 240)	97.6 (24168)	96 (24, 624)	96 (7, 624)
Hemodialysis/hemofiltration (N)	1 (6.3%)	11 (15.3%)	0	2 (16.7%)	2 (15.4%)	16 (12.7%)

Abbreviations: BCAA, branched chain amino acid; IV, intravenous.

^a^ Multiple choice questions.

^b^ Two cases of neonatal episode, two cases of physical efforts, one travel abroad, Burkitt lymphoma, one abdominal pains, and one anemia possibly responsible for decompensation.

Three episodes were excluded because baseline leucine was <381 μmol/L. The evaluable sample was 126 episodes from 54 patients, of these five episodes were excluded because they could not be stratified by treatment route. The majority (n = 49, 90.7%) were diagnosed after identification by newborn screening before 2 months of age and presented a classic MSUD phenotype (n = 46, 85.2%).

### 3.1 Episode characteristics and management

Of the 126 episodes, febrile illness/infection was the most common cause of metabolic decompensation (n = 51, 40.5%), followed by dietary issues, including non‐compliance to dietary restrictions (n = 28, 22.2%) and surgery or planned surgery (n = 8, 6.3%). The majority of metabolic decompensations (n = 85, 67.5%) were accompanied by clinical signs at hospital admission, of which “gastrointestinal disorders” (n = 43, 34.1%) and “nervous system disorders” (n = 40, 31.7%) were the most common. Leucine levels at baseline were mildly (n = 51, 40.5%), moderately (n = 47, 37.3%), severely (n = 13, 10.3%) and critically (n = 15, 11.9%) elevated.

All decompensation episodes required emergency treatments (n = 126, 100%) with protein intake being discontinued (n = 114, 90.5%) or reduced (n = 12, 9.5%) and administration of BCAA‐free formula (n = 125, 99.2%). Most commonly, BCAA‐free formula was administered along with glucose/dextrose (n = 101, 80.2%) and/or lipids (n = 82, 65.1%) resulting in a high caloric treatment (n = 84, 66.7%). In approximately one‐third of episodes (37.6%) multiple administration routes for BCAA‐free formula were employed (also within the same episode) The most common route of administration (as percentage of total administrations) for BCAA‐free formula was nasogastric tube (n = 69, 55.2%; “enteral”), followed by oral (n = 60, 48%), IV (n = 51, 40.8%), and gastrostomy (n = 2, 1.6%). Reasons for administration of IV BCAA‐free formula included gastric intolerance (n = 17, 33.3%), refusal of nasogastric tubing (n = 16, 31.4%), “emergency” (n = 7, 13.7%), coma (n = 4, 7.8%), and as prophylaxis before a planned surgery (n = 3, 5.9%). In order to maintain the BCAA balance, valine and isoleucine were re‐introduced in a median time of 3.0 days after onset of the decompensation episode, followed by leucine (median time: 5.5 days).

### 3.2 Oral/enteral and IV BCAA‐free formula analysis

At baseline, mean (±SD) leucine plasma concentrations were 791.4 μmol/L (±267.7) for the oral/enteral group and 921.1 μmol/L (±333.5) for the IV group. Leucine levels at baseline did not appear to be evenly distributed across groups. In the oral/enteral group they were predominantly (92.8%) mild or moderately elevated, and in 7.2% of cases severe or critically elevated. In the IV group, 25% of cases were severe or critically elevated and 75% of cases were mild or moderately elevated (Table 2). Enteral or oral BCAA‐free formula was frequently used in children (94%, adults: 44.4%) and the IV formulation was frequently used in adults (85.2%, children: 21.7%).

**TABLE 2:** Leucine levels by treatment route stratifications^a^

	Oral/enteral group	IV group
Number of episodes	69 (54.8%)	36 (28.6%)
Leucine level at admission (μmol/L)		
N	69 (100%)	36 (100%)
Mean (SD)	791.4 (267.7)	921.1 (333.5)
Median (range)	757 (388.8, 1721)	908 (382, 1684)
Leucine category at admission		
Mild (5.0 mg/dL (381 μmol/L) ≤[Leu] <10.0 mg/dL (762 μmol/L))	36 (52.2%)	10 (27.8%)
Moderate (10.0 mg/dL (762 μmol/L) ≤[Leu] <15.0 mg/dL (1144 μmol/L))	28 (40.6%)	17 (47.2%)
Severe (15.0 mg/dL (1144 μmol/L) ≤[Leu] <20.0 mg/dL (1525 μmol/L))	3 (4.3%)	8 (22.2%)
Critical ([Leu] ≥20.0 mg/dL (1525 μmol/L))	2 (2.9%)	1 (2.8%)
Mean (SD) leucine level day 1 (μmol/L)	617.0 (252.3)	755.4 (251.6)
Mean (SD) leucine level day 2 (μmol/L)	446.7 (247.2)	584.5 (293.7)
Mean (SD) leucine level day 3 (μmol/L)	348.1 (229.5)	386.2 (279.3)
Mean (SD) leucine level day 4 (μmol/L)	335.9 (233.8)	289.6 (222.1)
Mean (SD) leucine level day 5 (μmol/L)	320.4 (230)	358 (280.1)
Mean (SD) leucine level day 6 (μmol/L)	307.8 (225.1)	379.2 (310.9)
Mean (SD) leucine level day 7 (μmol/L)	279.8 (197.9)	293.7 (121.9)
Mean (SD) leucine level based on last leucine reported (μmol/L)^b^	242.9 (163.8)	263.9 (145.6)

^a^ Data for excluded episodes or for patients who received HD/HF not shown.

^b^ Day at discharge was different between patients.

**FIGURE 1:**
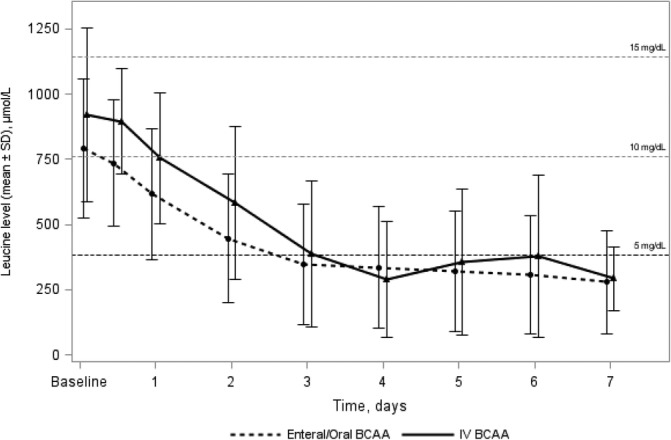
Evolution of leucine levels over the first 7 days by treatment route

The mean (±SD) time to first leucine normalization (<381 μmol/L) was 68.4 hours (±52.9) in the oral/enteral group and 95.6 hours (±79.2) in the IV group. The evolution of leucine levels in the oral/enteral group compared with those of the IV group during the first 7 days of treatment are shown in Figure 1. Overall, leucine normalization at discharge was achieved in 76.8% (n = 53) of episodes in the oral/enteral group and in 80.6% (n = 29) in the IV group. At discharge, leucine levels had decreased by 69.3% in the oral/enteral group (mean leucine level at baseline:  μmol/L, mean leucine level at discharge: 242.9 μmol/L) and by 71.3% in the IV group (mean leucine level at baseline: 921.1 μmol/L, mean leucine level at discharge: 263.9 μmol/L).

Except for one patient per group, all decompensations were resolved at time of discharge. Mean (±SD) time to episode resolution was 9.2 days (±6.2) in the oral/enteral group and 7.4 days (±4.6) in the IV group (*P* = NS) (Table 3). The mean (±SD) length of hospital stay was 6.6 days (±4.2) days for the oral/enteral group and 5.4 days (±2.7) for the IV group (*P* = NS). The mean (±SD) length of ICU stay was 3.2 days (±2.1) for the oral/enteral group and 2.5 days (±1.3) for the IV group (*P* = NS).

**TABLE 3:** Clinical outcomes according to group stratifications^a^

Outcome	Oral/enteral group (N = 69)	IV group (N = 36)
Time to episode resolution (days)^b^		
N	67 (98.5%)^c^	35 (100%)
Mean (SD)	9.2 (6.2)	7.4 (4.6)
Median (range)	7 (2, 35.0)	6 (2, 19)
Normalization of leucine		
Yes	55 (79.7%)	29 (80.6%)
No	13 (18.8%)	7 (19.4%)
Unknown	1 (1.4%)	0
Time to normalization of leucine (hours)^d^		
N	55 (100%)	29 (100%)
Mean (SD)	68.4 (52.9)	95.6 (79.2)
Median (range)	48.8 (6.1, 334.3)	72 (23.9, 428.8)
Length of hospital stay (days)		
Mean (SD)	6.6 (4.2)	5.4 (2.7)
Median (range)	5 (2, 23)	5 (2, 14)
Was ICU/RU stay required?		
No	63 (91.3%)	32 (88.9%)
Yes	6 (8.7%)	4 (11.1%)
Length of ICU stay (days)		
N	6 (100%)	4 (100%)
Mean (SD)	3.2 (2.1)	2.5 (1.3)
Median (range)	2.5 (1, 7)	2.5 (1, 4)

^a^ Episodes treated with HD/HF (N = 16) are not shown.

^b^ Episodes without end date were not included in the time to episode resolution analysis. Ongoing episodes were censored.

^c^ Missing N: 1 (1.5%).

^d^ Episodes that did not reach normalization were censored. Only one episode was excluded from the time to normalization analysis as only two plasma leucine values were reported on the same day without associated time. Normalization is the first‐time leucine reaches values <381 μmol/L.

### 3.3 Age‐category analysis

**FIGURE 2:**
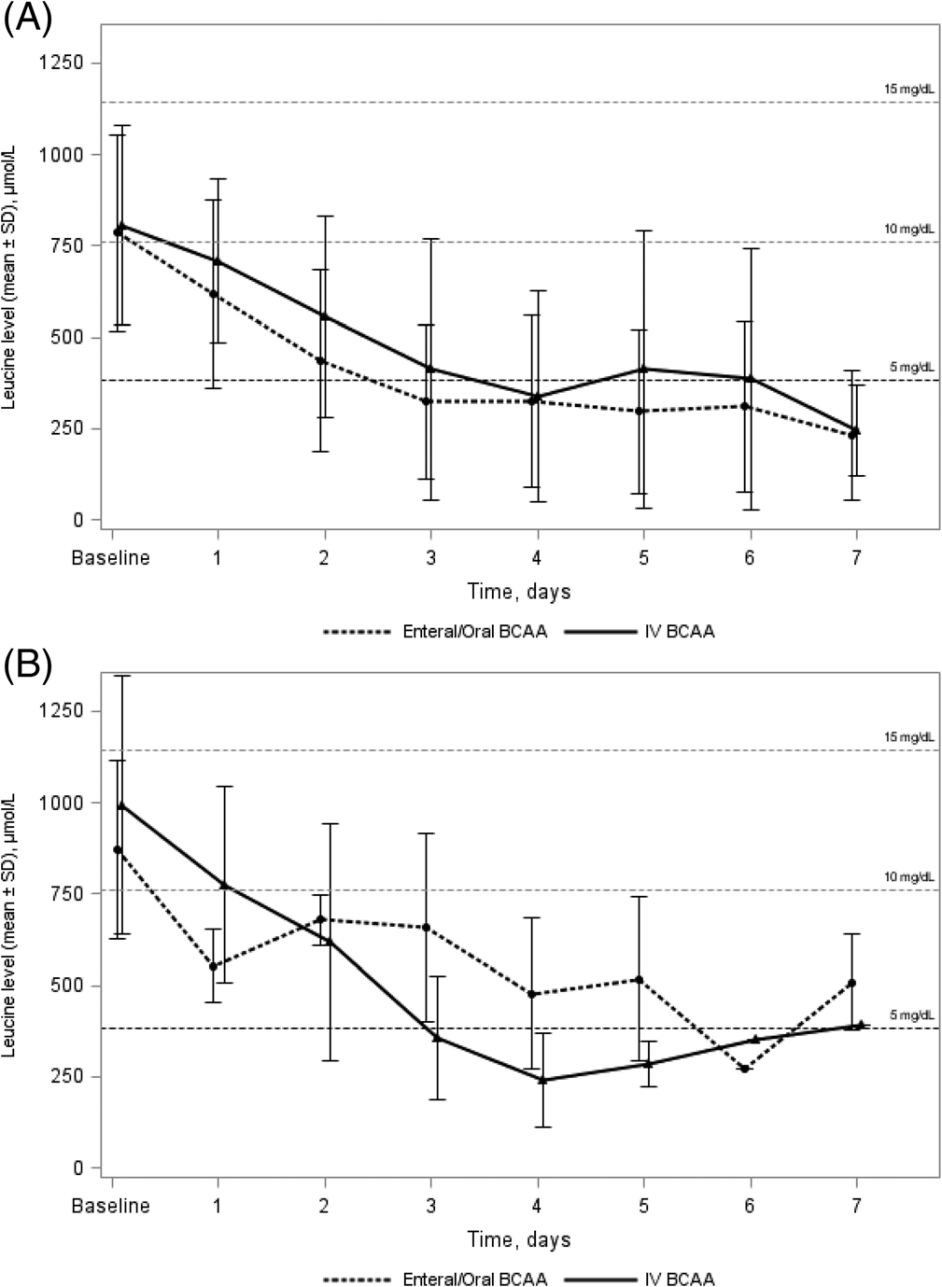
**A,** Evolution of leucine levels stratified by treatment group over the first 7 days in the pediatric population. **B,** Evolution of leucine levels stratified by treatment group over the first 7 days in the adult population

**FIGURE 3:**
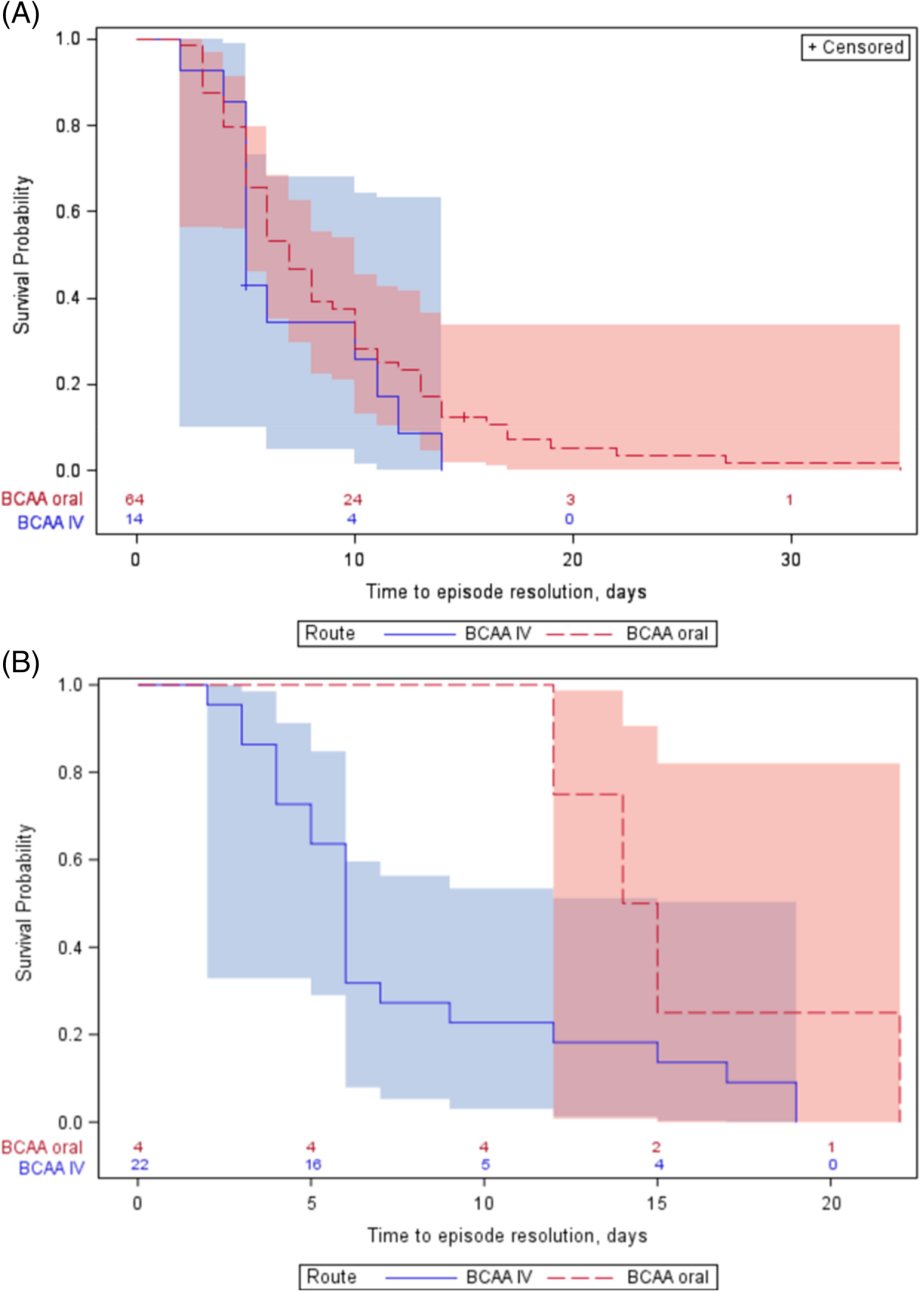
**A,** Kaplan‐Meier analysis of time to episode resolution for pediatric patients based on treatment received. **B,** Kaplan‐Meier analysis of time to episode resolution for adult patients based on treatment received

In the subpopulation of pediatric patients, reduction in leucine levels over time for both treatment groups is shown in Figure 2A. The mean (±SD) leucine level at baseline was 786.3 μmol/L (±270) in the oral/enteral group (n = 65) and 806.9 μmol/L (±272.3) in the IV group (n = 14). In the oral/enteral subgroup, leucine levels at baseline were graded as mild 53.8%, moderate 38.5%, severe 4.6%, and critical 3.1%; while in the IV subgroup, they were mild 42.9%, moderate 42.9%, and severe 14.3%. Results for the pediatric groups (oral/enteral vs IV) were: percentage reaching normalization (83.1%, n = 54 vs 85.7%, n = 12); mean (±SD) time to first leucine normalization (68.3 hours [±53.4] vs 84.1 hours [±59.8]); and mean (±SD) time to episode resolution (8.8 days (±6) vs 6.8 days (±3.6), *P* = NS). In the Kaplan‐Meier analysis (Figure 3A), median (95% CI) time to episode resolution was 7 days (6–9) for the oral/enteral group and 5 days (5–11) for the IV group (log‐rank test *P* = .24). The duration of hospitalization was the same in both treatment groups (mean 6.6 days).

In the subpopulation of adults, reduction in leucine levels over time for both treatment groups is shown in Figure 2B. The mean (±SD) leucine level at baseline for the oral/enteral group (n = 4) was 873 μmol/L (±244.3), with leucine levels graded as mild 25% and moderate 75%; whereas in the IV group (n = 22), the mean (±SD) leucine level at baseline was 993.8 μmol/L (±353.9), with leucine levels graded as mild 18.2%, moderate 50%, severe 27.3%, and critical 4.5%. Leucine normalization at discharge was reached in one of four episodes (25%) in the oral/enteral group and 17 of 22 episodes (77.3%) in the IV group. Mean (±SD) time to first leucine normalization was 72.8 hours (±0.0) for the oral/enteral group and 103.8 hours (±91.4) for the IV group. Conversely, mean time (±SD) to episode resolution was 15.8 days (±4.3) in the oral/enteral group and 7.7 days (±5.2) in the IV group (*P* = .008). In the Kaplan‐Meier analysis (Figure 3B), median (95% CI) time to episode resolution was 14.5 days (12‐22) for the oral/enteral group and 6 days (4–7) for the IV group (log‐rank test; *P* = .063). Mean duration of hospitalization (±SD) was 6 days (±2.6) for the oral/enteral group and 4.6 days (±1.7) for the IV group; the median (range) was 6 days (4, 8) and 4 days (3, 5) respectively (*P* = NS).

### 3.4 Safety analysis

Seven serious adverse events (SAEs) in two patients were reported during hospitalization. These included fever, soreness, nausea, headache, abdominal pain, and vomiting. Only nausea and vomiting were treatment‐related, due to enteral BCAA‐free formula being poorly tolerated. No SAEs were reported in the IV group. All events were resolved.

## 4 DISCUSSION

Although some management recommendations have been published,^15^ there is limited evidence of real‐world management of MSUD decompensation episodes. This study showed that across France and Germany there is heterogeneity in treatment approaches. Although in the vast majority of cases, treatment involved discontinuation of proteins and administration of a BCAA‐free formula, the availability of the IV product created unavoidable variations in care. The real‐world data reported here suggest that there are several challenging clinical conditions that might require the IV administration of BCAA‐free formula. These include gastric intolerance or refusal to nasogastric tubing, the patient's clinical condition (eg, comatose patients), or in the event of surgery.

Both the oral and the IV BCAA‐free formulations were found to be clinically effective, achieving leucine normalization in approximately 2.8 days in the oral group and 4 days in the IV group. Leucine normalization at discharge was achieved in approximately 80% in both groups. The IV BCAA‐free formula was comparably effective as the oral/enteral administration in reducing leucine levels. At discharge, the mean decrease in leucine levels of 548.5 μmol/L (69.3%) in the oral/enteral group and 657.2 μmol/L (71.3%) in the IV group may have been because baseline leucine levels were 791.4 μmol/L for the oral/enteral group and 921.1 μmol/L for the IV group.

With the caveat that the differences in outcomes between the oral/enteral and IV groups for the overall population were not statistically different, it is nevertheless noteworthy that the mean “time to leucine normalization” was around 2.9 days for the oral/enteral group and 4 days in the IV group, but the mean “time to episode resolution” was around 9.2 days in the oral/enteral group and 7.4 days in the IV group. This apparent but albeit not significant difference may have resulted from the “time to episode resolution” variable encompassing both leucine normalization and/or the resolution of clinical signs and symptoms as determined by the attending physician, which may have biased these findings. But it is because this outcome includes clinically meaningful elements, we believe it is a useful outcome for clinicians treating decompensation episodes. The short time to episode resolution reported with the IV BCAA‐free formula may have implications for hospital resource use.

The Kaplan‐Meier analyses provided descriptive statistical comparisons of the administration routes in the pediatric and adult subpopulations. Although, neither pediatric nor adult analysis reached statistical significance, there is arguably a trend in the adult subpopulation (*P* = .063) for “time to episode resolution” favoring the IV BCAA‐free formula. This comparison has a major limitation in that the adult subpopulation receiving oral/enteral BCAA‐free formula was only four patients. The contributing factors may have been a relatively high mean leucine level at baseline in the adult population, where the majority of episodes were graded as “moderate,” whereas in the pediatric group, the majority were graded as “mild.” A clinical explanation for these high leucine levels at baseline may have been because of relatively delayed hospitalizations compared with children, who are generally rushed to hospital. In the pediatric group, all outcomes for the oral/enteral and the IV groups were numerically very similar. However, in the adult group, the mean time to episode resolution was approximately 8 days shorter in the IV group than in the oral/enteral group (*P* = .008). This result is all the more surprising given the relative severity of the disease at baseline in the IV group. Given the small population sizes in the adult subgroup, these findings will need to be further investigated.

Both oral/enteral and IV BCAA‐free formulas were safe and well tolerated. Seven adverse events were reported that met the severity criteria, of which only two were considered associated with the oral/enteral BCAA‐free formula. These were instances of nausea in one patient and vomiting in another as a result of enteral nutrition not being tolerated.

The limitation of this study is that it is a retrospective analysis of medical records and was restricted to a descriptive analysis of treatments received and outcomes reported, which allowed for a certain heterogeneity within groups, in terms of treatments (eg, the IV BCAA‐free formula was not available at the German center at the time) and in terms of definitions of outcomes, all of which would be expected to introduce a degree of bias. The inclusion of episodes involving HD/HF may have been another source of bias; they were relevant to the descriptive analysis of the population as a whole, but as they would be expected to have a significant contribution to clinical and biochemical outcomes, they were excluded from the exploratory analysis of BCAA‐free formulas. Similarly, subjective and heterogeneous definitions of “episode resolution” and the use of different laboratory methodologies for measuring leucine levels may also have been sources of bias. As this was a retrospective study, standardized measurement timepoints could not be predefined. Another limitation was that the administration route was variable for each episode, allowing a different subsequent route during the same episode, but given the retrospective nature of the study, this was unavoidable. This factor was a particular limitation in the current study as individuals assigned to the IV group might also have been switched to receive oral/enteral BCAA‐free amino acid substitutions in the course of the respective decompensations. While this was indeed a significant study limitation, it was unavoidable in real‐world management as physicians may have preferred to introduce oral formulas subsequent to IV administration as a prelude to discharge.

The particular strength of this study is that it is the largest cohort study in MSUD yet conducted, building on a previous analysis,^16^ to provide greater insight into the optimal management of decompensation episodes and offers further confirmation of the clinical efficacy, safety, and utility of the IV BCAA‐free treatment for use in emergency settings.

## 5 CONCLUSIONS

This descriptive analysis shows the burden that MSUD decompensation episodes place, not only on patients but also on healthcare systems. It suggests that although both orally/enterally or IV administered BCAA‐free formula are highly effective in reducing neurotoxic levels of leucine, for adults, the IV formula tends to be associated with a shorter time to episode resolution. Moreover, the safety and tolerability profile appears to be favorable in all populations for both the oral/enteral and the IV solution, with no SAEs reported for the IV solution. Considering that excessive leucine levels are a clinical emergency condition, IV administration of BCAA‐free formula may be a particularly useful alternative in emergency settings, if the patient is not able to receive or cannot tolerate oral treatment or enteral tubing.

## CONFLICTS OF INTEREST

Pascale de Lonlay, Roland Posset, Ulrike Mütze, Karine Mention, Delphine Lamireau, Manuel Schiff, Aude ServaisS, Jean Baptiste Arnoux, Anaïs Brassier, Myriam Dao, Claire Douillard, Chris Ottolenghi, and Clément Pontoizeau have no conflicts of interest. Federica Miotto and Jeannie Le Mouhaër are employees of Recordati Group.

## ETHICS STATEMENT

This was a retrospective noninterventional study. All patients or their legal guardians gave informed consent or assent to the use of their anonymized data.

## Supporting information


**Table S1** Nutritional information of IV branched‐chain amino‐acids‐free amino‐acid formula
