# Supplementary material for: Real‐world management of maple syrup urine disease (MSUD) metabolic decompensations with branched chain amino acid‐free formulas in France and Germany: A retrospective observational study
Source: JIMD Rep. 2021 Mar 6;59(1):110–9. doi: 10.1002/jmd2.12207 (PMC8100389; doi:10.1002/jmd2.12207)
Supplement: Supplementary file 1 — Table S1 Nutritional information of IV branched‐chain amino‐acids‐free amino‐acid formula [file JMD2-59-110-s001.docx]

**Supplement 1.**

Table 1: Nutritional information of IV branched-chain amino-acids-free amino-acid formula

| **Ingredients** | **Average content per 1000 mL** |
| --- | --- |
| Energy | 200 Kcal |
| Amino acids | 52 g |
| Alanine | 6.3 g |
| Arginine | 4.1 g |
| Aspartic acid | 4.1 g |
| Cysteine | 1.0 g |
| Glutamic acid | 7.1 g |
| Glycine | 2.1 g |
| Histidine | 2.1 g |
| Lysine | 5.6 g |
| Methionine | 1.3 g |
| Phenylalanine | 2.7 g |
| Proline | 5.6 g |
| Serine | 3.8 g |
| Taurine | 0.3 g |
| Threonine | 3.6 g |
| Tryptophan | 1.4 g |
| Tyrosine | 0.5 g |
